# DNA Replication Dynamics of the GGGGCC Repeat of the *C9orf72* Gene*

**DOI:** 10.1074/jbc.M115.660324

**Published:** 2015-10-13

**Authors:** Ryan Griffin Thys, Yuh-Hwa Wang

**Affiliations:** From the Department of Biochemistry and Molecular Genetics, University of Virginia, Charlottesville, Virginia 22908

**Keywords:** amyotrophic lateral sclerosis (ALS) (Lou Gehrig disease), DNA, DNA replication, DNA structure, genomic instability

## Abstract

DNA has the ability to form a variety of secondary structures in addition to the normal B-form DNA, including hairpins and quadruplexes. These structures are implicated in a number of neurological diseases and cancer. Expansion of a GGGGCC repeat located at *C9orf72* is associated with familial amyotrophic lateral sclerosis and frontotemporal dementia. This repeat expands from two to 24 copies in normal individuals to several hundreds or thousands of repeats in individuals with the disease. Biochemical studies have demonstrated that as little as four repeats have the ability to form a stable DNA secondary structure known as a G-quadruplex. Quadruplex structures have the ability to disrupt normal DNA processes such as DNA replication and transcription. Here we examine the role of GGGGCC repeat length and orientation on DNA replication using an SV40 replication system in human cells. Replication through GGGGCC repeats leads to a decrease in overall replication efficiency and an increase in instability in a length-dependent manner. Both repeat expansions and contractions are observed, and replication orientation is found to influence the propensity for expansions or contractions. The presence of replication stress, such as low-dose aphidicolin, diminishes replication efficiency but has no effect on instability. Two-dimensional gel electrophoresis analysis demonstrates a replication stall with as few as 20 GGGGCC repeats. These results suggest that replication of the GGGGCC repeat at *C9orf72* is perturbed by the presence of expanded repeats, which has the potential to result in further expansion, leading to disease.

## Teaser

**Background:** The (GGGGCC)_n_ hexanucleotide repeat expansion of *C9orf72* is the most common genetic cause of ALS-FTD.

**Results:** *C9orf72* repeat expansion increases instability and decreases replication efficiency by disrupting replication fork progression.

**Conclusion:** *C9orf72* repeat length and replication direction contribute to repeat instability in human cells.

**Significance:** DNA replication-induced instability at the *C9orf72* GGGGCC repeat can lead to further expansion and more severe disease.

## Introduction

The expansion of a GGGGCC hexanucleotide repeat sequence located in a noncoding region of the *C9orf72* gene was discovered several years ago (1, 2) and is the most common genetic cause of amyotrophic lateral sclerosis (ALS)^2^ and frontotemporal dementia (FTD) (3, 4). Alleles of unaffected individuals contain between two and 24 GGGGCC repeats, with the majority of alleles having two repeats (5). In patients with ALS-FTD characterized by expanded repeats, the expansions can range from tens to thousands of repeats in length (1, 2). However, no distinct disease-causing threshold of repeat number has been identified.

Over 30 human diseases, mostly neurological and muscular, are associated with expansion of repetitive DNA sequences (6). Repetitive DNA sequences have the ability to form a variety of secondary structures during normal cellular processes such as replication and transcription because of the unwinding of duplex DNA. Although the mechanism for repeat expansion is still not fully understood, the ability of the repeat to form a stable secondary structure is critical for instability. The presence of structure-forming sequences on template or nascent DNA strands can potentially lead to contractions or expansions, respectively (7). G-quadruplex structures have the potential to influence instability because of their interaction with replication (8, 9) and transcription (reviewed in Ref. 10) machinery. The GGGGCC repeat at *C9orf72* can form stable DNA and RNA quadruplexes with as few as four repeats (11–13). Recent work has suggested that formation of RNA:DNA hybrids known as R-loops play a role in promoting repeat instability (14). Processing of the R-loops formed during transcription can lead to slipped DNA intermediates with the potential to result in expansions or contractions (14). These results may explain the genesis of repeat instability in non-replicating cells through transcriptional processes.

However, expansion may occur in earlier progenitor cells during development through a replication-mediated process. Studies to investigate the instability of trinucleotide repeats have led to the proposal of an “ori switch” model for repeat instability where firing of different replication origins can change the direction of replication through expanded repeats, leading to instability (7, 15–24). In a study of the CGG repeat associated with fragile X syndrome, Gerhardt *et al.* (16) have found that, in unaffected individuals, replication of the (CGG)_n_ repeat at the *FMR1* locus occurs equally in both directions. Replication originating upstream of the gene leads to CGG repeat contraction, whereas replication originating downstream of the gene leads to expansions, resulting in a balancing of contraction and expansion events (16). In fragile X embryonic stem cells, replication predominantly originates from the downstream of the gene, resulting in expansion (16). These results suggest that orientation of the replication origin relative to the repeat sequence is a predominant factor in causing instability.

In this study, we investigate the influence of GGGGCC repeat length and orientation relative to the replication origin in replication-induced instability. Using an SV40 replication system in HEK293T cells, we demonstrate a length-dependent increase in replication-dependent instability (both expansions and contractions) and show that repeat orientation relative to replication origin affects the propensity for expansions or contractions. Furthermore, we find that overall replication is decreased in a repeat length-dependent manner. The presence of 20 or more GGGGCC repeats caused replication fork stalling, as observed by two-dimensional gel electrophoresis. These results suggest that GGGGCC repeat instability occurs during replication and that the level and type of instability are dependent on repeat length and orientation.

## Experimental Procedures

### 

#### 

##### Plasmids

**FIGURE 1.**
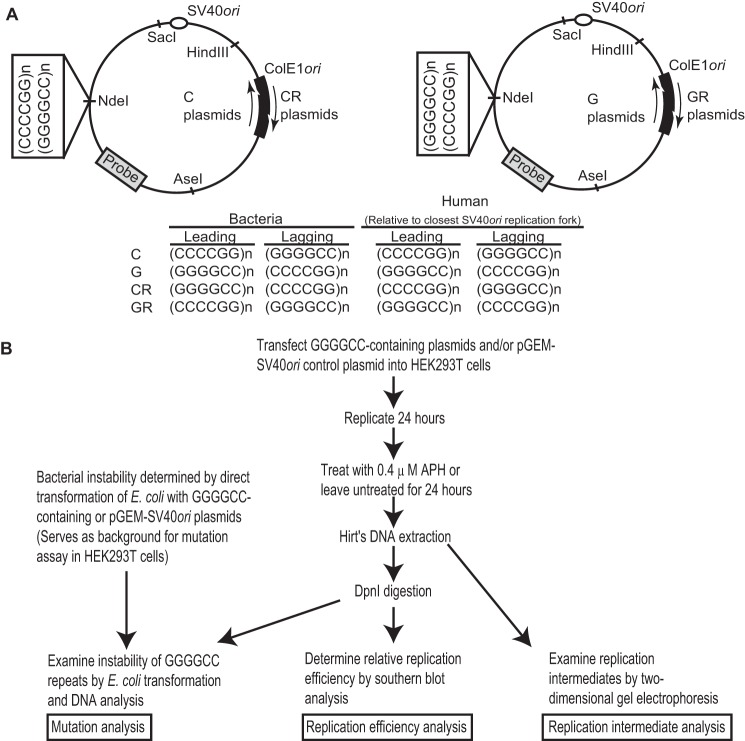
**Experimental designs for investigating DNA replication-induced GGGGCC repeat instability in bacteria and human cells.** *A*, plasmid maps showing the location of bidirectional SV40 origin of replication (*SV40ori*); (GGGGCC)_n_ repeats where *n* = 11, 21, or 41; and bacterial origin of replication (*ColE1ori*). Also shown are the restriction enzyme sites used in the experiments and the hybridization probe used for Southern blotting (see “Experimental Procedures”). Plasmid maps depict C and CR plasmids (*left panel*), and G and GR plasmids (*right panel*). Clones with an additional R designation are distinguished from their counterpart clones with the opposite orientation of ColE1*ori. Bottom panel*, table showing which repeat sequence, (GGGGCC)_n_ or (CCCCGG)_n_, serves as the leading strand and lagging strand templates for each plasmid set during replication in bacteria and human cells. For human replication, the orientation of the repeat listed is for the replication fork moving in the counterclockwise direction from the bidirectional SV40*ori* because this is the closest replication fork to the repeat sequence. *B*, experimental design for our studies investigating the effects of GGGGCC repeat length and orientation on DNA replication by examining instability, relative replication efficiency, and replication fork progression during replication of GGGGCC repeats. *APH*, aphidicolin.

Plasmids containing the GGGGCC repeats were created using a pGEM-SV40*ori* plasmid described previously (25). Inserts of varying size and orientation were created by annealing (GGGGCC)_n_ and (CCCCGG)_n_ oligonucleotides. Annealed duplexes were filled in using DNA polymerase I, large (Klenow) fragment (New England Biolabs) to generate blunt-ended, double-stranded fragments of varying length and then cloned into the NdeI-digested and blunt-ended pGEM-SV40*ori* plasmid to generate GGGGCC-containing plasmids (clones 11G, 11C, 20G, 22C, 41G, and 41C; Fig. 1*A*). Plasmids were named according to the number of repeats and the sequence composition of the leading strand template relative to the closest replication fork from the bidirectional SV40 *ori.*

For constructing clones 21CR and 41CR, which contain a ColE1*ori* in the opposite orientation to clones 22C and 41C, we removed the bacterial plasmid replication origin ColE1*ori* from clone 41C by restriction enzyme digestion with EcoP15I and AflIII (New England Biolabs). The ColE1*ori*-containing fragment was blunt-ended and religated to the rest of the 41C plasmid. Plasmid isolation was used to screen for clones containing the ColE1*ori* in the opposite orientation relative to clones 22C and 41C. This cloning procedure resulted in clones containing either 21 or 41 GGGGCC repeats with a C-rich leading strand (relative to the SV40*ori*) and a ColE1*ori* in the opposite orientation to clones 22C and 41C. The same cloning strategy was applied to the 41G clone to generate the 41GR clone which contains 41 GGGGCC repeats with a G-rich leading strand (relative to the SV40*ori*) and a ColE1*ori* in the opposite orientation to clone 41G. Clones with an additional “R” designation are distinguished from their counterpart clones with the opposite orientation of ColE1*ori* (Fig. 1*A*).

##### Replication Efficiency

Replication efficiency experiments were performed as described previously (25) with modifications (Fig. 1*B*). HEK293T cells were grown to 50% confluence before co-transfection with 800 ng of pGEM-SV40*ori* and 800 ng of each GGGGCC-containing plasmid using the CaPO_4_ method. To determine replication efficiency, SV40-replicated DNAs were digested with HindIII and NdeI (New England Biolabs) to linearize and distinguish the GGGGCC-containing plasmids from the pGEM-SV40*ori* control plasmid and with DpnI (New England Biolabs) to remove unreplicated parental templates. Southern blot analysis was then used to identify replicated DNA using an [α-^32^P]dCTP-labeled probe hybridizing to nucleotide numbers 1725–2132 of pGEM-SV40*ori*, which is present in all constructs. Replication efficiency was determined by the ratio of replicated GGGGCC-containing DNA to the pGEM-SV40*ori* control DNA using ImageQuant version 5.2 to measure the intensity of each band. Student's *t* test was performed to determine statistically significant differences between clones.

##### Mutation Assay

**FIGURE 2.**
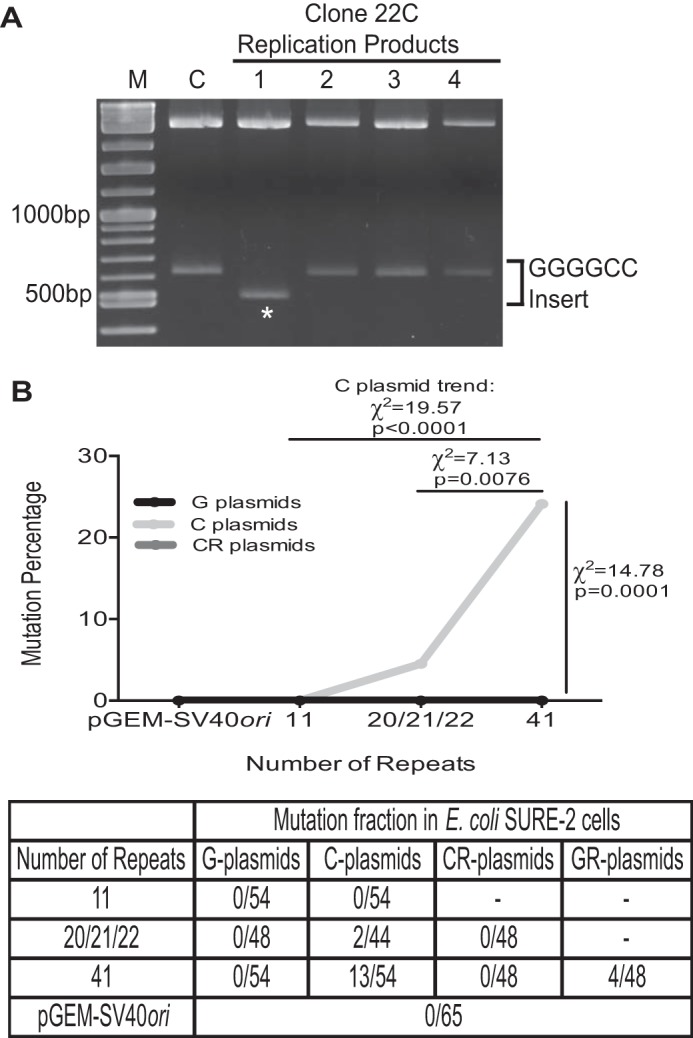
**C9orf72 hexanucleotide repeat instability in bacterial cells.** *A*, a representative agarose gel showing individual *E. coli* replication products from clone 22C. *Lanes 1–4*, individual *E. coli* replication products from clone 22C. The band of DNA containing the GGGGCC insert is indicated. *Lane 1* shows a contraction event as represented by the faster migrating band (*asterisk*) relative to the unreplicated DNA control (*lane C*). *Lane M*, molecular weight marker. *B*, quantification of instability in *E. coli* SURE2 cells. Plasmid DNA was transformed into SURE2 cells, and at least 44 individual colonies from each plasmid were picked at random for analysis. DNA was purified from selected colonies and digested with restriction enzymes to release the GGGGCC insert. Digested samples were resolved on agarose gels to compare with unreplicated controls as in *A*. The data are plotted as the percentage of mutations (contractions) of the total colonies analyzed. The table displays all results of the mutation assay as the number of mutations divided by the number of total colonies analyzed. Clone 41C is significantly more unstable during *E. coli* replication relative to clone 22C (χ^2^ = 7.13, *p* = 0.0076) and clone 41G (χ^2^ = 14.78, *p* = 0.0001). For C plasmids, there is a length-dependent increase in instability, as determined by χ^2^ test for trend (χ^2^ = 19.57, *p* < 0.0001).

**FIGURE 3.**
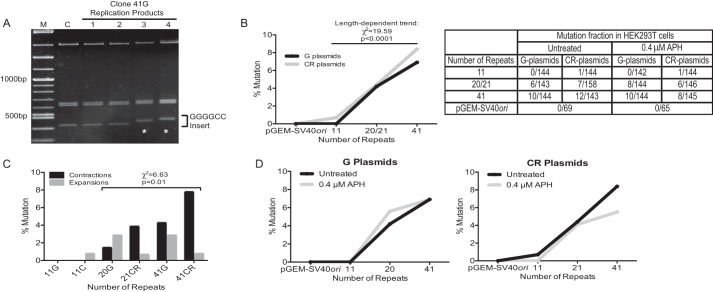
**Replication-induced instability in HEK293T cells.** *A*, representative agarose gel showing individual replication products from the replication of clone 41G in HEK293T cells. *Lanes 1–4*, individual HEK293T replication products from clone 41G. The band of DNA containing the GGGGCC insert is indicated. Replication products 3 and 4 exhibit repeat expansions, as determined by the slower migrating bands (*asterisks*) relative to the unreplicated DNA control (*lane C*). Sanger DNA sequencing revealed that products 3 and 4 contain 44 and 46 repeats, respectively. *Lane M*, molecular weight marker. *B*, *left panel*, quantification of G (*black line*) and CR (*gray line*) plasmid instability in HEK293T cells. Mutation percentage is measured as the number of mutations relative to the number of colonies analyzed. *Right panel*, the table displays full results of the mutation assay as the number of mutations divided by the number of total colonies analyzed. Instability increased in a length-dependent manner for G plasmids (χ^2^ = 9.85, *p* = 0.007), CR (χ^2^ = 9.91, *p* = 0.007) plasmids, and for both combined (χ^2^ = 19.59, *p* < 0.0001). *APH*, aphidicolin. *C*, quantification of contractions (*black columns*) or expansions (*gray columns*) following replication in HEK293T cells. The types of mutations generated by replication of the plasmids with 20–41 repeats were significantly different (χ^2^ = 6.63, *p* = 0.01) because expansions were more likely to result from replication of G plasmids (8 of 10 total), and contractions were more likely the result of CR plasmid replication (17 of 25 total). *D*, quantification of G (*left panel*) and CR (*right panel*) plasmid instability in HEK293T cells after no treatment (*black lines*) or 24-h treatment with 0.4 μm aphidicolin (*gray lines*).

Instability of GGGGCC-containing constructs was examined using a modified stability of trinucleotide repeat by individual product assay (Fig. 1*B*) as described previously (25). Briefly, products of replication from transfected HEK293T cells were digested with DpnI to eliminate unreplicated parental templates and transformed into SURE2 cells (Stratagene). Single colonies from each clone were selected at random (see Fig. 3*B* for the numbers of colonies analyzed), and DNAs were isolated using the GeneJET plasmid miniprep kit (Thermo Scientific) and digested with appropriate restriction enzymes to release the GGGGCC-containing insert. Samples were run on 1.3% agarose gels and scored for insertion or deletion events, as determined by slower or faster migrating bands, respectively, relative to unreplicated DNA controls. Bacterial instability was examined by directly transforming *Escherichia coli* SURE2 cells with unreplicated plasmid DNA before scoring for mutation events (see Fig. 2*B* for the numbers of colonies analyzed). Mutations were verified by Sanger DNA sequencing of the repeat-containing insert to determine the number of repeats deleted or inserted. A χ^2^ test was used to determine the statistical significance of instability among replication products. The χ^2^ test for trend was used to determine the statistical significance for the linear relationship between repeat length and instability.

##### Two-dimensional Gel Electrophoresis (2DGE)

HEK293T cells were grown to 50% confluence in 100-mm-diameter dishes before transfection with 5 μg of plasmid DNA. Cells were allowed to grow, following transfection, for 16 h before replacing the medium. Plasmid replication intermediates were extracted from cells by Hirt method, followed by treatment with proteinase K, phenol/chloroform extraction, and alcohol precipitation. Purified DNA was digested with AseI and SacI restriction enzymes (New England Biolabs). 2DGE was performed by electrophoresis of the DNA in the first dimension on 0.4% agarose gel for 16 h at 26 V. The lanes containing separated DNA intermediates were then excised, rotated 90°, and embedded in 1% agarose gel containing 0.5 μg/μl ethidium bromide. The second dimension was run at 156 V for 5 h at 4 °C. Gels were subjected to Southern blotting as described in the replication efficiency experiments. Quantification of the replication intermediates was determined using ImageQuant version 5.2 to measure the intensity of 13 equal segments along the Y-arc of each image, with segment one being located at the 1n location and segment 13 being located at the 2n location. Each segment is shown as percent of the total intensity of the 13 segments to account for fluctuations between autoradiogram exposures. Student's *t* test on the average intensities of each segment was performed to determine statistically significant differences between clones.

## Results

### 

#### 

##### Generation of GGGGCC Repeat-containing Plasmids

To investigate whether expansion of GGGGCC repeats affects DNA replication, we utilized an SV40 replication system as described previously (25). Repeats of varying lengths and orientations were introduced into plasmids containing an SV40 origin of replication (SV40*ori*) (Fig. 1*A*). These plasmids varied in length with 11, 20 (22), or 41 repeats and the G-rich (GGGGCC) strand serving as either the leading strand or lagging strand template relative to the closest replication fork from the bidirectional SV40*ori*. Plasmids were named according to the number of repeats and the sequence composition of the leading strand template (*i.e.* clone 11G refers to a plasmid containing the (GGGGCC)_11_ sequence on the leading strand template). With triplet repeat diseases such as fragile X syndrome, a threshold of disease has been vetted previously. However, for the GGGGCC repeat at *C9orf72*, no such threshold has been identified for any of the diseases for which the expansion is implicated as causative. The number of repeats investigated in our study represents the number of repeats found on unaffected alleles (11 or 20 (22) repeats) or a length outside the normal range (41 repeats), allowing us to investigate any phenotypic differences between “normal” repeat lengths and expansions outside the range found in unaffected individuals. We generated six GGGGCC-containing plasmids (clones 11G, 11C, 20G, 22C, 41G, and 41C) and one control plasmid (pGEM-SV40*ori*), which contains the SV40*ori* but lacks any GGGGCC repeats.

##### Replication of GGGGCC Repeats Causes Repeat Contractions in Bacteria Depending on Repeat Length and Orientation Relative to Replication Origin

During cloning of the GGGGCC-containing plasmids, we noticed a high level of instability because of bacterial DNA replication, even with the use of *E. coli* SURE2 cells designed to reduce instability. To investigate this instability, we transformed SURE2 cells with individual plasmids and allowed the bacteria to grow for 6 h before selecting individual colonies and isolating plasmid DNA (Fig. 1*B*). Purified plasmid DNA was digested with appropriate restriction enzymes to release the GGGGCC insert. Inserts were analyzed by agarose gel electrophoresis to identify expansions or contractions, as determined by slower or faster migrating bands, respectively (Fig. 2*A*), followed by Sanger DNA sequencing to determine the number of repeats. At least 44 individual colonies for each clone were analyzed, and the instability is quantified in Fig. 2*B*. Clones 22C and 41C were mutated in 4.5% or 24.1% of colonies, respectively, whereas the pGEM-SV40*ori* control plasmid and clones 11G, 11C, 20G, and 41G displayed no instability (Fig. 2*B*). Clone 41C, representing a repeat length outside of the normal range seen in unaffected individuals, was significantly more unstable than clones 22C (χ^2^ = 7.13, *p* = 0.0076) and 41G (χ^2^ = 14.78, *p* = 0.0001). All mutations identified for clones 22C and 41C were contractions, similar to previous studies of trinucleotide repeat instability in bacteria (26). The C-plasmids, 11C, 22C, and 41C, displayed a significant relationship between repeat length and instability (χ^2^ = 19.57, *p* < 0.0001), whereas the G-plasmids did not cause mutation. These studies demonstrate that repeat orientation relative to the bacterial plasmid replication origin (ColE1*ori*) contributes to repeat instability in a repeat length-dependent manner. During ColE1*ori* replication, clones 22C and 41C, which have GGGGCC repeats located on the lagging strand template, could have G-quadruplex structure formation on the lagging strand template, leading to repeat contractions, with longer repeats possibly forming a more stable structure.

To determine the importance of GGGGCC repeat orientation relative to ColE1*ori* in plasmid instability, we created plasmids containing 21 or 41 repeats similar to clones 22C and 41C but with the ColE1*ori* in the opposite direction, resulting in clones 21CR and 41CR, respectively (Fig. 1*A*). During replication of these clones in bacteria, the replication fork encounters the GGGGCC repeats in the same orientation as clones 20G and 41G, which render CCCCGG repeats on the lagging strand template. Indeed, neither clone 21CR nor clone 41CR displayed any instability (Fig. 2*B*), similar to clones 20G and 41G, but in contrast to unstable clones 22C and 41C, demonstrating that orientation of the GGGGCC repeats on the lagging strand template of the replication fork drives instability in bacteria (as seen in clones 22C and 41C). To test this further, we created clone 41GR, which is similar to clone 41G but with the ColE1*ori* in the opposite direction. As expected, the level of 41GR instability was significantly higher than clone 41G (χ^2^ = 4.68, *p* = 0.030), again showing that the GGGGCC sequence on the lagging strand template promotes instability. Interestingly, replication of clone 41GR resulted in significantly less instability than of clone 41C (χ^2^ = 4.53, *p* = 0.033). Clones 41GR and 41C have the same GGGGCC sequence as the lagging strand template, but the intervening sequences from the ColE1*ori* to the repeat sequence in these two clones is different, potentially contributing to the difference in instability in 41GR and 41C. However, we found that clone 41GR was significantly more unstable than 41CR (χ^2^ = 4.17, *p* = 0.041), which shares the same intervening sequence and differs only in the repeat orientation. The high level of instability for clones 41C and 41GR demonstrates the toxicity of the expanded repeats in bacteria, even in cells designed to reduce instability. These results suggest that expanded GGGGCC repeats on the lagging strand template during bacterial plasmid replication leads to GGGGCC repeat contractions, possibly because of the formation of G-quadruplex structures.

##### Replication-induced Repeat Expansions and Contractions in Human Cells Occur in a Length- and Orientation-dependent Manner

Next, a mutation assay was performed to determine the effects of GGGGCC repeat length and orientation on instability during replication in HEK293T cells (Fig. 1*B*). Individual replication products were examined using a modified stability of trinucleotide repeat by individual product assay, as described previously (25). Briefly, purified DNA from transfected HEK293T cells was digested with DpnI to remove unreplicated DNA products and transformed into *E. coli* SURE2 cells. Each of the replication products was analyzed by agarose gel electrophoresis to identify expansions or contractions, as determined by slower or faster migrating bands, respectively (Fig. 3*A*), followed by Sanger DNA sequencing to determine the number of repeats. Because pGEM-SV40*ori* and clones 11G, 11C, 20G, 21CR, 41G, and 41CR showed no instability in SURE2 cells, we can ascertain that the level of GGGGCC repeat instability observed in the mutation assay is due to DNA replication of human cells. As shown in Fig. 3*B*, although the pGEM-SV40*ori* control, clone 11G, and clone 11C showed no significant instability, replication through longer repeats led to an increase in mutation events. DNA replication through clones 20G and 21CR, representing the high end of the unaffected patient allele repeat length, led to mutations in 4.2% and 4.4% of all colonies analyzed, respectively (Fig. 3*B*). Furthermore, replication of clones 41G and 41CR increased mutations to 6.9% and 8.4% of colonies analyzed, respectively. Importantly, instability occurred in a length-dependent manner for G plasmids (χ^2^ = 9.85, *p* = 0.007), CR plasmids (χ^2^ = 9.91, *p* = 0.007), and both combined (χ^2^ = 19.59, *p* < 0.0001).

Analysis of all mutations during replication of plasmids with 20–41 repeats found that 71% (25 of 35) were repeat contractions and 29% were repeat expansions (Fig. 3*C*). Although there was no significant difference between the overall levels of mutation between the G and CR plasmids, the types of mutations generated by replication of these plasmid sets was significantly different (χ^2^ = 6.63, *p* = 0.01) because expansions were more likely to result from replication of G plasmids (8 of 10 total), and contractions were more likely the result of CR plasmid replication (17 of 25 total). Interestingly, all contractions and expansions identified were the result of loss or gain, respectively, of intact GGGGCC repeats, as verified by Sanger DNA sequencing. The longest expansion is one 46-repeat mutant from clone 41G. DNA replication-induced expansions occurred more frequently in 20G and 41G, whereas contractions occurred more frequently in 21CR and 41CR, suggesting that the presence of the G-rich sequence on the nascent lagging strand is more susceptible to expansion, whereas the presence of the G-rich sequence on the lagging strand template is more susceptible to contraction.

##### Perturbation of the Replicative DNA Polymerases Does Not Affect Instability of GGGGCC Repeats

To investigate the role of the replicative polymerase in DNA replication-induced instability, the mutation assay was performed in the presence of low-dose aphidicolin, an inhibitor of the DNA replicative polymerases α, δ, and ϵ. Treatment of transfected HEK293T cells with 0.4 μm aphidicolin for 24 h did not significantly affect the instability of the repeat sequences regardless of repeat length and replication orientation (Fig. 3*D*). Overall, the levels of instability were similar to those seen in untreated cells. Treatment with aphidicolin caused repeat contraction in most cases (29 of 33). These data suggest that replication stress has little effect on DNA replication-induced GGGGCC repeat instability in human cells.

##### GGGGCC Repeats Decrease Overall Plasmid Replication Efficiency

**FIGURE 4.**
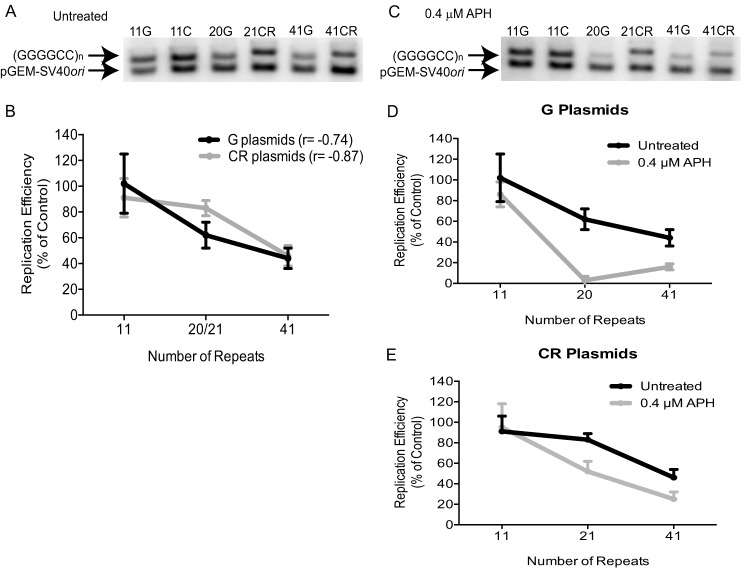
**Replication efficiency of GGGGCC repeats in HEK293T cells.** *A*, representative Southern blot showing the replicated DNA of plasmids containing GGGGCC repeats (*top band*) or pGEM-SV40*ori* control (*bottom band*). *B*, quantification of replication efficiency of each GGGGCC-containing plasmid. The replication efficiency is determined by the ratio of the GGGGCC-containing plasmid DNA band relative to the pGEM-SV40*ori* control DNA band. Results are shown as the mean ± S.D. from at least three individual experiments for each plasmid. Pearson correlation coefficients were calculated for G plasmids (*r* = −0.74) and CR plasmids (*r* = −0.87). *C*, representative Southern blot showing the replicated DNA after treatment of HEK293T cells with 0.4 μm aphidicolin (*APH*) for 24 h. *D* and *E*, quantification of G (*D*) and CR (*E*) plasmid replication efficiency upon treatment with aphidicolin. Results are shown as the mean ± S.D. from at least three individual experiments for each plasmid.

The mutation analysis results suggest that DNA replication has difficulty progressing through GGGGCC repeats, leading to instability. To examine how GGGGCC repeats affect completion of DNA replication in human cells, the replication efficiency of GGGGCC-containing plasmids was determined by co-transfecting the repeat-containing plasmid and the pGEM-SV40*ori* control plasmid into HEK293T cells (Fig. 1*B*). Cells were allowed to grow for 48 h before low molecular weight viral DNA was purified by the Hirt method. Purified DNA was then digested with DpnI so that only fully replicated plasmid DNA was present. The DNA was also digested with restriction enzymes to linearize the plasmids and distinguish the GGGGCC-containing plasmids from the control. Following Southern blot analysis, the intensity of the GGGGCC-containing and the control plasmid bands, representing fully replicated DNA, was measured (Fig. 4*A*). The relative replication efficiency of each GGGGCC-containing plasmids compared with the control plasmid was determined as the ratio of the amount of fully replicated GGGGCC-containing plasmid to the amount of fully replicated pGEM-SV40*ori* control plasmid. As shown in Fig. 4, *A* and *B*, DNA replication efficiency decreases in a length-dependent manner (Pearson correlation coefficient, *r* = −0.74 for G plasmids; *r* = −0.87 for CR plasmids). The replication efficiency of clones 11G and 11C was similar to the pGEM-SV40*ori* control plasmid, as demonstrated by replication efficiency values of 102% ± 23% and 91% ± 15%, respectively. Replication of clones 20G and 41G decreased to 62% ± 10% and 44% ± 8% efficiency relative to pGEM-SV40*ori*, respectively (Fig. 4*B*). Similarly, clones 21CR and 41CR demonstrated a reduction in replication efficiency to 83% ± 6% and 46% ± 8%, respectively (Fig. 4*B*). Replication efficiency decreased in a length-dependent manner for both G plasmids and CR plasmids, suggesting that repeat length affects the completion of DNA replication of GGGGCC sequences in human cells.

To investigate the effect of GGGGCC repeats on DNA polymerase in completing replication, we performed the replication efficiency analysis in the presence of 0.4 μm aphidicolin (Fig. 4, *C–E*). Low-dose aphidicolin significantly reduced the replication efficiency of clones 20G (3% ± 4%) and 41G (16% ± 3%) compared with that of the untreated cells (*p* = 2.6 × 10^−14^ and *p* = 2.4 × 10^−5^, respectively, Fig. 4*D*). In the presence of aphidicolin, a significant reduction was also observed in the replication efficiency of clones 21CR (52% ± 10%) and 41CR (25% ± 7%) relative to that of untreated cells (*p* = 3.0 × 10^−5^ and *p* = 2.4 × 10^−8^, respectively, Fig. 4*E*). Comparison of the effect on opposing orientations found that clones 20G and 41G demonstrated a significantly lower replication efficiency relative to their matched samples (21CR and 41CR, *p* = 3.1 × 10^−9^ and *p* = 0.002, respectively; Fig. 4, *D* and *E*). These results suggest that replication stress affects the relative DNA replication efficiency of GGGGCC repeats and displays a stronger influence in G plasmids than in CR plasmids.

##### Replication Forks Stall at the Site of Expanded GGGGCC Repeats

**FIGURE 5.**
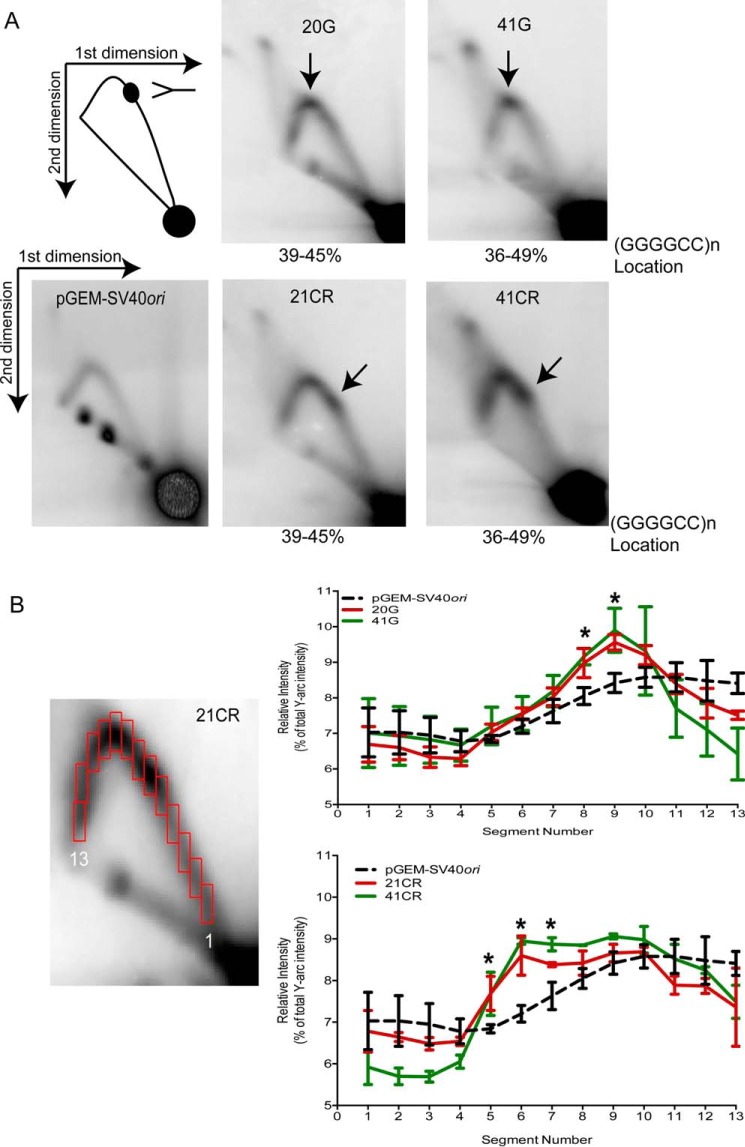
**Replication fork stalling at GGGGCC repeats in HEK293T cells visualized by two-dimensional gel electrophoresis.** *A*, schematic showing an arc containing Y-shaped replication intermediates and a block in replication fork progression. Shown are representative 2DGE images for pGEM-SV40*ori* control and GGGGCC-containing plasmids. *Arrows* point to replication fork stalls during DNA replication of clones 20G, 21CR, 41G, and 41CR in HEK293T cells. The percentages below each image refer to the relative distance of the GGGGCC insert from the SV40*ori* to the total length of the plasmid fragment analyzed by 2DGE. *B*, representative 2DGE image of 21CR (*left panel*) showing the method used for quantification of the intensity of the single Y arc (see “Experimental Procedures”). The single Y arc was divided into 13 segments of equal size, and the intensity of each segment was measured, with segment one being located at the 1n location and segment 13 being located at the 2n location. The relative intensities (percent of total intensity of a single Y arc) of replication intermediates for each segment are plotted (*right panel*) for pGEM-SV40*ori* and G-plasmids (*top panel*) or CR-plasmids (*bottom panel*). Results are shown as the mean ± S.D. from at least three individual 2DGE images for each plasmid. *, *p* < 0.002 compared with pGEM-SV40*ori*.

To examine how the replication fork progresses through GGGGCC repeats, we identified replication intermediates formed during replication in HEK293T cells using 2DGE (Fig. 1*B*). The location of the restriction enzymes used in our analysis (Fig. 1*A* and “Experimental Procedures”) allowed us to visualize Y-shaped replication fork intermediates formed during plasmid replication. Analysis of the pGEM-SV40*ori* control plasmid replication intermediates after 24 h of replication revealed the expected pattern of an arc containing Y-shaped intermediates (Fig. 5*A*), as predicted by the location of the restriction enzyme sites. DNA replication of all GGGGCC repeat-containing plasmids with 20 or more repeats (20G, 21CR, 41G, and 41CR) demonstrated a replication fork stall, as indicated by an accumulation of replication intermediates at the estimated location of the GGGGCC repeats (Fig. 5*A*, *arrows*). In contrast, replication of clone 11G, clone 11C (data not shown), and the pGEM-SV40*ori* control did not contain an apparent accumulation of replication intermediates. To quantify the extent of replication fork stalling for all plasmids, we divided the single-Y arc into 13 segments of equal size and measured the intensity of each segment relative to the total intensity of all 13 segments (Fig. 5*B*). Quantification of these results found that the relative intensity at the estimated site of GGGGCC repeats for 20G and 41G (segment numbers 8 and 9) was significantly higher than the intensity of the same location for pGEM-SV40*ori* (*p* < 0.002, Fig. 5*B*). Also, the relative intensity at the site of GGGGCC repeats for 21CR and 41CR (segment numbers 5–7) was significantly higher than the intensity of the same location for pGEM-SV40*ori* (*p* < 0.002, Fig. 5*B*). Interestingly, there was no significant difference between the relative intensity at the site of replication fork stalling between 20G and 41G (*p* = 0.5) or between 21CR and 41CR (*p* = 0.3). These results suggest that the presence of at least 20 GGGGCC repeats has the ability to stall replication fork progression in human cells.

## Discussion

Here, for the first time, we demonstrated the ability of the GGGGCC repeat to interfere with DNA replication in human cells. When replication of GGGGCC-containing plasmids could be completed, we found that instability occurred in a length-dependent manner. Instability increased with the number of GGGGCC repeats for both G-rich and C-rich leading strand plasmids. We also found an orientation-dependent effect on the propensity for these repeats to expand or contract. The GGGGCC repeat expansion is more prone to occur when the structure-forming G-rich sequence is found on the nascent lagging strand, as seen for clones 20G and 41G. Repeat contraction is more prone to occur when the structure-forming G-rich sequence serves as the lagging strand template, as seen for clones 21CR and 41CR. Previous studies have shown that the replication fork orientation, as it encounters a tract of structure-forming repeat DNA, influences the propensity for the repeat DNA to be expanded or contracted, leading to the proposal of the ori switch model of repeat expansion (7, 15–18). In this model, replication through an expanded repeat can lead to contractions or expansions. Replication from either direction can occur at the same locus so that expansions and contractions work against each other to keep the repeat tract at a relatively constant, although fluctuating, length (27). Inactivation of either replication origin can shift the balance of expansions and contractions, potentially leading to repeat expansion. Our data suggest that the presence of the quadruplex structure-forming G-rich sequence on the nascent lagging strand, as for clones 20G and 41G, is more prone to causing repeat expansion in human cells, possibly because of strand slippage resulting from the formation of DNA secondary structure. On the other hand, quadruplex structure formation on the lagging strand template, as possible for clones 21CR and 41CR, could result in DNA polymerase bypass of the structure and lead to repeat contraction.

The GGGGCC repeat length also affects DNA replication efficiency through these repeat regions because the amount of fully replicated plasmids decreased in a length-dependent manner. In the absence of replication stress, there is no difference in overall replication between plasmids with G-rich or C-rich leading strand templates. However, upon treatment with low-dose aphidicolin, we found an orientation-dependent effect on replication. Perturbation of the replicative polymerase almost completely blocked replication of clones 20G and 41G in HEK293T cells, whereas replication of clones 21CR and 41CR was decreased to a lesser extent. These results suggest that, for GGGGCC repeats, the ability to form stable quadruplex structure can affect DNA replication. The presence of the quadruplex-forming G-rich sequence on the lagging strand template (as in 21CR and 41CR) can tolerate for the DNA polymerase to bypass the structure and allow, to some extent, the completion of DNA replication. However, the presence of the quadruplex structure on the nascent lagging strand (as in 20G and 41G) has the potential to cause replication slippage and can affect the ability of the polymerase to complete DNA replication.

Our results are the first to demonstrate that replication fork progression stalls at the site of GGGGCC repeats in human cells. This stalling was evident with as few as 20 repeats and occurred independent of repeat orientation relative to the replication fork. Interestingly, the level of replication fork stalling did not increase with the length of GGGGCC repeats. The resolution power of our 2DGE assay may not be sufficient to accurately measure the differences in replication intermediate accumulation between 20 and 41 repeats.

Repeat expansion is associated with a number of diseases, including developmental and neurological disorders (17). The ability of these repeats forming stable secondary structure is well documented, such as CTG and CGG repeats to form stable hairpin structures (18, 28–30), GAA repeats to form triplex DNA (31), and CGG repeats to form quadruplex structures (32). The ability of these structures to interfere with normal cellular processes, such as DNA replication and transcription, is a contributing factor to their expansion. Our results are consistent with previous studies investigating the stability of similarly GC-rich CGG repeats during DNA replication in a variety of organisms. CGG repeats are able to stall the replication fork in bacteria, yeast, and primate cells (23, 33, 34). Additionally, the orientation of CGG repeats has been shown to be an important factor in stability during DNA replication (19–24).

Our results demonstrate a repeat length-dependent mechanism for GGGGCC repeat instability and replication efficiency in human cells. Longer repeats increase instability, as measured by contraction and expansion events, and decrease replication efficiency, possibly because of the formation of more stable quadruplex structures that result in a more pronounced phenotype. The replication orientation affects the propensity for expansions or contractions, which supports the ori switch model, a mechanism proposed for trinucleotide repeat expansion. Our studies provide a mechanism for *C9orf72* instability in which the repeat length and replication direction through the GGGGCC repeat contribute to the level and type of repeat instability.

## Author Contributions

R. G. T. designed, performed, and analyzed the experiments. Y. H. W. conceived and coordinated the study. Both authors wrote the manuscript.

## Footnotes

2 The abbreviations used are:

ALS: amyotrophic lateral sclerosis
FTD: frontotemporal dementia
2DGE: two-dimensional gel electrophoresis.
